# Transferrin-modified liposomes triggered with ultrasound to treat HeLa cells

**DOI:** 10.1038/s41598-021-90349-6

**Published:** 2021-06-02

**Authors:** Nour M. AlSawaftah, Nahid S. Awad, Vinod Paul, Paul S. Kawak, Mohammad H. Al-Sayah, Ghaleb A. Husseini

**Affiliations:** 1 Department of Chemical Engineering, American University of Sharjah, PO. Box 26666, Sharjah, UAE; 2 Department of Biology, Chemistry and Environmental Sciences, American University of Sharjah, PO. Box 26666, Sharjah, UAE

**Keywords:** Cancer, Drug discovery

## Abstract

Targeted liposomes are designed to target specific receptors overexpressed on the surfaces of cancer cells. This technique ensures site-specific drug delivery to reduce undesirable side effects while enhancing the efficiency of the encapsulated therapeutics. Upon reaching the tumor site, these liposomes can be triggered to release their content in a controlled manner using ultrasound (US). In this study, drug release from pegylated calcein-loaded liposomes modified with transferrin (Tf) and triggered with US was evaluated. Low-frequency ultrasound at 20-kHz using three different power densities (6.2 mW/cm^2^, 9 mW/cm^2^ and 10 mW/cm^2^) was found to increase calcein release. In addition, transferrin-conjugated pegylated liposomes (Tf-PEG liposomes) were found to be more sonosensitive compared to the non-targeted (control) liposomes. Calcein uptake by HeLa cells was found to be significantly higher with the Tf-PEG liposomes compared to the non-targeted control liposomes. This uptake was further enhanced following the exposure to low-frequency ultrasound (at 35 kHz). These findings show that targeted liposomes triggered with US have promising potential as a safe and effective drug delivery platform.

## Introduction

Cancer is a worldwide health problem, with the last few decades witnessing a rapid increase in cancer cases. The American Cancer Society reported that 1,806,590 new cancer cases and 606,520 cancer deaths are projected to occur in 2020 in the United States alone^1^. Chemotherapeutic drugs are among the most effective methods to treat cancer as they disseminate throughout the body, destroying cancer cells and reducing tumor volumes. However, most of these anti-neoplastic agents are insoluble or poorly soluble in water. They are highly toxic and lack the ability to differentiate between cancerous and healthy cells. This causes several adverse side effects that reduce the patients’ quality of life, including hair loss, fatigue, anemia, nausea, and cardiotoxicity.

Nanocarriers are nanoscale particles (1–1000 nm) capable of encapsulating drugs and safely delivering them to the tumor site. The unique nanoscale properties of these carriers and specific bio-functions allow them to accumulate in specific targeted regions and release their payloads in a controlled fashion hence increasing the drug targeting efficiency and reducing side effects^2^. Furthermore, these nanocarriers improve the solubility and lifetime of the drug in the blood, in addition to improving their therapeutic indices^3^. Various nanocarriers have been successfully developed for drug delivery applications, including carbon nanotubes (CNTs), mesoporous silica nanoparticles (MSNs), gold nanoparticles (GNPs), super magnetic iron oxide nanoparticles (SPIONs) and Quantum dots (QD), liposomes, polymeric micelles (PMs), dendrimers, and solid lipid nanoparticles (SLNs)^4^. The unique properties of these nanocarriers allow them to passively target cancer cells due to their ability to extravagate through the leaky vasculature surrounding the tumor and accumulate in large numbers in diseased tissues. This phenomenon is known as the enhanced permeability and retention (EPR) effect^5–7^. Unfortunately, poor drug release and drug efflux pumps result in sub-lethal drug concentrations and the possible development of multidrug resistance (MDR) at the diseased targeted site^8^. Therefore, encapsulated drugs must be released in a fast and controlled manner to provide an effective dose capable of destroying the cancer cells before they acquire the ability to circumvent the therapeutic effect of these drugs^9^. Conventional nanocarriers can be modified or functionalized to be responsive to specific triggering techniques. The triggering stimulus could be either endogenous (e.g., pH, temperature, redox, ionic environment, and enzymatic level) or exogenous (e.g., temperature, light, magnetic field, electric field and ultrasound)^10–15^.

Liposomes are bilayer spherical nanovessels prepared from naturally occurring phospholipids. These phospholipids consist of a fatty acid-based hydrophobic tail (forming the hydrophobic bilayer) and a phosphate-based hydrophilic head facing the external aqueous environment and hydrophilic core. Liposomes can efficiently encapsulate both hydrophilic drugs, inside their cores, and hydrophobic agents inside the lipid bilayer. To overcome the rapid clearance of these drug carriers by the reticuloendothelial systems (RES), liposomes have been coated with polymers, most notably polyethylene glycol (PEG), i.e., pegylation, forming “stealth” liposomes^16^. Although pegylation provides liposomes with increased stability and longer circulation times, conventional liposomes lack specificity and selectivity. To address this issue, liposomal surfaces can be modified with targeting moieties that bind to specific receptors overly expressed on the surfaces of cancer cells. Different types of targeting moieties have been used, including proteins, peptides, antibodies, carbohydrates, glycoproteins, and vitamins^17,18^.

**Figure 1:**
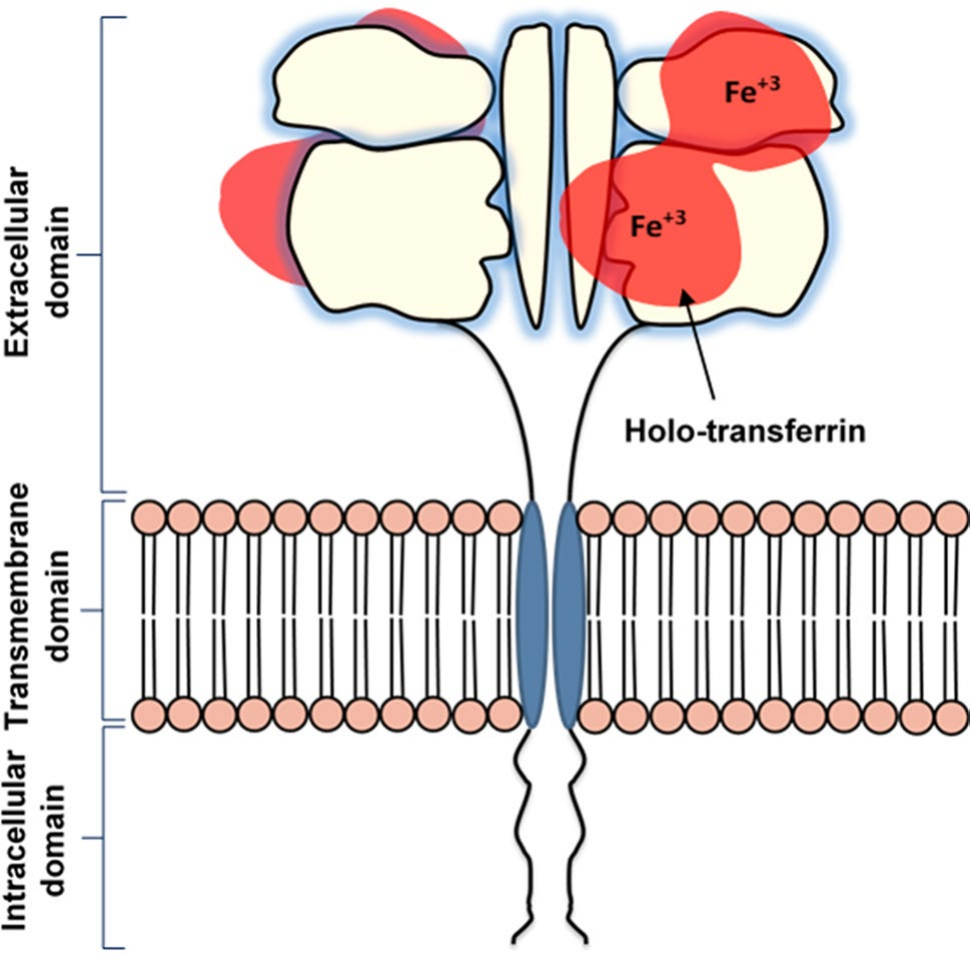
Schematic diagram of holo-transferrin binding to transferrin receptor. Regenerated from^17^.

Transferrin (Tf), the moiety of choice in this research, is a double-lobed serum glycoprotein, with a molecular weight of approximately 80 kDa and a polypeptide chain containing 679 amino acids, including two specific high-affinity Fe(III) binding sites^19^. The Transferrin receptor family includes TfR1 (also known as CD71) and TfR2 (also known as CD77)^20,21^. Tf is a natural iron-transport glycoprotein primarily secreted by the liver. However, iron is not naturally produced by the body, and hence is obtained from our diet. Tf is responsible for regulating the transport and cellular uptake of iron via TfR1-mediated endocytosis. Iron-free Tf (apo-transferrin) can bind to two iron molecules, forming holo-transferrin. Holo-transferrin binds to TfR1 receptors, as shown in Fig. 1.

Cancer cells are defined by their rapid cell proliferation rate, leading to a higher iron demand used in DNA synthesis and cell cycle progression. This intrinsically high demand for iron leads to the overexpression of Tf receptors on the surfaces of hematopoietic malignancies and solid tumors such as liver, prostate, breast, and ovarian cancers^20,22,23^. The high expression of TfR1 by cancer cells makes it an attractive route for targeted drug delivery in cancer therapy. The employment of TfR1 can be achieved by encapsulating chemotherapeutic drugs inside targeted liposomes crafted with the targeting molecule (Tf) to target Tf receptors; thus improving tumor targeting, drug specificity, and therapeutic efficacy while reducing systemic toxicity.

Several studies have investigated the effect of Tf conjugation on liposomal delivery of chemotherapeutic drugs. For example, Tf-modified liposomes loaded with vincristine and tetrandrine^24^ and cisplatin^25^ showed higher levels of accumulation and anticancer activity in glioma-bearing mice compared to free drugs. An earlier study by Li et al.^26^ demonstrated that Tf-modified stealth liposomes significantly enhanced tumor uptake of doxorubicin (DOX) and thus, improved its therapeutic activity against liver cancer. Tf-modified liposomes were also found to increase the cytotoxicity of docetaxel by 3.6-fold compared to the unmodified control liposomes^27^.

To further explore the concept of triggering and controlling the rate of drug release from Tf-modified liposomes, US will be utilized to trigger calcein release from Tf-modified stealth (pegylated) liposomes (Tf-PEG-liposomes) in this work. Ultrasound (US) is used as a triggering mechanism for drug release from liposomes following their accumulation inside tumor tissues^28–31^. US consists of sound waves with frequencies higher than those of the audible range (> 20 kHz). Because of the mechanical nature of acoustic waves, they require a medium to propagate through^32^. These waves form a series of high pressure (compression) and low pressure (rarefaction) regions. When traveling through a liquid medium, the energy produced from these compressions and rarefactions creates microscopic gas bubbles in the medium (i.e., acoustic cavitation). As the waves progress, these gas bubbles oscillate forward and backward, either in a slow manner where the bubble’s radius varies about an equilibrium value (stable cavitation) or in a rapid manner where the bubble expands to two- or three-folds their resonant size and collapse vigorously (i.e. inertial/collapse cavitation). During inertial cavitation, the collapse of bubbles produces surges in local temperature and pressure in the cavitation spots (at the microscopic level) reaching temperatures of 5000 K and extremely high pressures. US produces two main effects: mechanical effects (i.e. cavitation) and thermal effects^33,34^. The acoustic effects depend on the frequency and intensity of US and can be employed to trigger drug release from liposomes. Since liposomes consist of phospholipid bilayers, they have similar structures to those of the human cell members, and thus cavitation will enhance both:

1. Drug release of anti-cancer agents from liposomes (following the nanoparticles’ accumulation at the tumor site) by inducing transient pores or pore-like defects in their membranes as shown by earlier studies^35,36^. Moreover, coating the liposomes with polymers (pegylation) was found to increase the liposomes’ sensitivity to low-frequency ultrasound (LFUS) compared to non-pegylated liposomes^28,37^.
2. Drug delivery to the cancer cells by creating pores in their cell membranes (i.e., sonoporation). Thus, increasing drug uptake by the diseased cells^38^.

Stable cavitation can release the contents of liposomes by disrupting cell membranes. Stable cavitation can also create microstreaming effects around the bubbles, causing pore formation. Inertial cavitation, on the other hand, creates strong shock waves with high pressures capable of disrupting the cell membrane and liposomal bilayer^39^. Both stable and inertial cavitation form reactive oxygen species (ROS), resulting in lipid peroxidation and membrane destruction^40^. In a previous study conducted by our group, we investigated the release kinetics of the model drug calcein from three different types of targeted liposomes, namely human serum albumin (HSA), Tf and arginylglycylaspartic acid (RGD), sonicated with LFUS (20-kHz). Our results showed that pegylated liposomes were more sonosensitive compared to non-pegylated liposomes; moreover, HSA-PEG and Tf-PEG liposomes showed higher calcein release following their exposure to pulsed LFUS compared to the control pegylated liposomes^41^. Targeted liposomes have a great potential in enhancing drug specificity and targeted drug delivery to the tumor. However, combining targeted liposomes with LFUS ensures controlled and efficient drug release at the diseased site temporally and spatially. US mediated release is expected to be higher using LFUS compared to high-frequency ultrasound (HFUS) because cavitation events are easier to induce under LFUS conditions. Cavitation is more likely to occur when bubbles are given enough time to grow and collapse, which is easier to achieve using LFUS because the time interval for the negative peak pressure is sufficient for nucleation^42^. In this study, calcein-loaded pegylated liposomes will be synthesized and conjugated to Tf molecules (Tf-PEG) to target cervical cancer cells by binding to the overexpressed Tf receptors on their surface. LFUS will then be applied to trigger calcein release from the liposomes, thus enhancing this model drug/dye's uptake by cancer cells. The application of LFUS will also result in sonoporation, whereby transient pores are formed in cell membranes, which will also enhance calcein uptake.

The quantitative analysis of the chemical, physical, and biological phenomena involved in drug release permits a better understanding of the mechanisms underlying drug release, which in turn would help improve the safety of the developed products^43–45^. The mathematical models used in this study are zero-order, first-order, Higuchi, Korsmeyer–Peppas, and Gompertz. The assumptions on which these models are based, their corresponding equations and linearizations are summarized in Table 2 (supplementary material).

## Materials and methods

### Materials

Phospholipids used to prepare the liposomes: 1,2-dipalmitoyl-sn-glycero-3-phosphocholine (DPPC) and 1,2-distearoyl-sn-glycero-3-phosphoethanolamine-*N*-[amino(polyethylene glycol)-2000] (ammonium salt) (DSPE-PEG(200)-NH2) were purchased from Avanti Polar Lipids Inc. (Alabaster, AL, USA, supplied by Labco LLC. Dubai, UAE). Calcein disodium salt, holo-transferrin human, cholesterol, the bicinchoninic acid kit, the RPMI-1640 media, and the fetal bovine serum were purchased from Sigma-Aldrich Chemie GmbH (supplied by Labco LLC. Dubai, UAE). The HeLa cell line was purchased from the European Collection of Authenticated Cell Cultures (ECACC general cell collection, Salisbury, UK).

### Preparation of control liposomes

Liposomes were prepared using the thin-film hydration method^46^. Briefly, liposomes were prepared using cholesterol, DPPC, and DSPE-PEG(2000)-NH_2_ at molar ratios of 30:65:5, respectively. The lipids were dissolved in 4 ml chloroform in a round-bottom flask. The chloroform was then evaporated using a rotary evaporator under vacuum at 50 °C for 15 min until a thin film was observed on the walls. Next, the lipid film was hydrated using 2 ml of a 50-mM calcein solution, and the pH was adjusted to 7.4. To obtain unilamellar vesicles, the solution was sonicated for 2 min using a 40-kHz sonication bath (Elma D-78224, Melrose Park, IL, USA). Whereas for particle size reduction, liposomes were extruded thirty times using 200-nm polycarbonate filters (Avanti Polar Lipids, Inc., Alabaster, AL, USA)^30,31,41,47^. The purification of the formulation involved gel exclusion chromatography using a Sephadex G-100 column (after equilibrating it with PBS (pH 7.4)). Finally, the collected fractions were stored at 4 °C. The encapsulated calcein concentration was estimated using a calibration curve of the fluorescence intensity versus different concentrations of calcein dissolved in PBS (pH 7.4). Calcein is self-quenched at high concentrations showing no fluorescent properties. At lower concentrations, calcein does not exhibit self-quenching.

### Preparation of Tf -PEG liposomes

**Figure 2:**
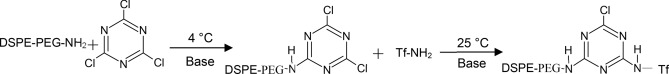
Transferrin (Tf) conjugation to the liposomes using cyanuric chloride as a coupling agent.

The functionalization of liposomes with transferrin (Tf) was performed using cyanuric chloride as a coupling agent . The liposomes were prepared using the procedure detailed above. As for the functionalization procedure, 10 mg of cyanuric chloride (2,4,6 trichloro-1,3,5 triazine) were dissolved in 500 µl of acetone and 2.5 ml of deionized water, which were added to the liposomes in a 1:1 molar ratio with the DSPE-PEG-NH_2_. The reaction was then kept at 0 °C for 3 h with gentle stirring before adding 0.125 ml of the human holo-transferrin protein (2 mg of transferrin dissolved in 1 ml of borate buffer at pH ~ 8.5), and the mixture was left stirring overnight. Tf-PEG liposomes were then passed through a G-100 PBS column (1 g of G-100 Sephadex in 20 ml of PBS at pH ~ 7.4) to remove any free proteins^41,47^. Figure 2 shows the conjugation reaction using cyanuric chloride as a coupling agent.

### Particle size determination

 The particle size and polydispersity of both targeted and non-targeted liposomes were measured with a dynamic light scattering instrument (DLS) using DynaPro, NanoStar (Wyatt Technology Corp., Santa Barbara, CA, USA). The sample to be measured was prepared by diluting 15 μl of liposomes in 1 ml of PBS^30,41,47^.

### Estimation of total phospholipid content

 The phospholipid content of the prepared liposomes was determined calorimetrically using the Stewart assay^48^. Liposomes were transferred to a round bottom flask and placed in the rotary evaporator under vacuum in order to dry up the medium in which the liposomes were suspended. One ml of chloroform was added to the flask, and this mixture was sonicated until no more particles were visible in the solution. Varying volumes of liposomes were pipetted out of the flask and transferred to centrifuge tubes. Chloroform was added to these tubes in such a way that the volume of liposomes, in addition to the volume of chloroform, would add up to 2 ml^30,31,41,47^. Two ml of ammonium ferrothiocyanate were then added to the tubes. The solutions in the tubes were mixed vigorously, followed by centrifugation for 10 min at 1000 rpm. The top dark layer was removed and discarded, while the bottom clear chloroform layer was removed and transferred to a quartz cuvette. The optical density of this chloroform phase was measured using ultraviolet–visible spectroscopy at A_max_ = 485 nm against chloroform as a blank. The procedure was repeated for transferrin-coupled liposomes, and three replicates were used for each sample.

### Protein quantification using the bicinchoninic acid (BCA) assay

The transferrin conjugation efficiency to liposomes was determined by the Bicinchoninic Acid Assay (BCA). The BCA reagent was prepared by mixing QuantiPro QA buffer, QuantiPro QB, and CuSO_4_ in a ratio of 25:25:1, respectively. The reagent was added to an Eppendorf tube (1 ml), and 600 ml of the PBS buffer and 400 μl of the liposomes were also added to the same tube followed by incubation at 60 °C for 60 min. UV–vis spectroscopy at A_max_ = 562 nm was used to measure the optical density of the samples. Three replicates were used for each experimental condition^30,31,41,47^.

### Measuring the power density of the ultrasonic probe

**Figure 3:**
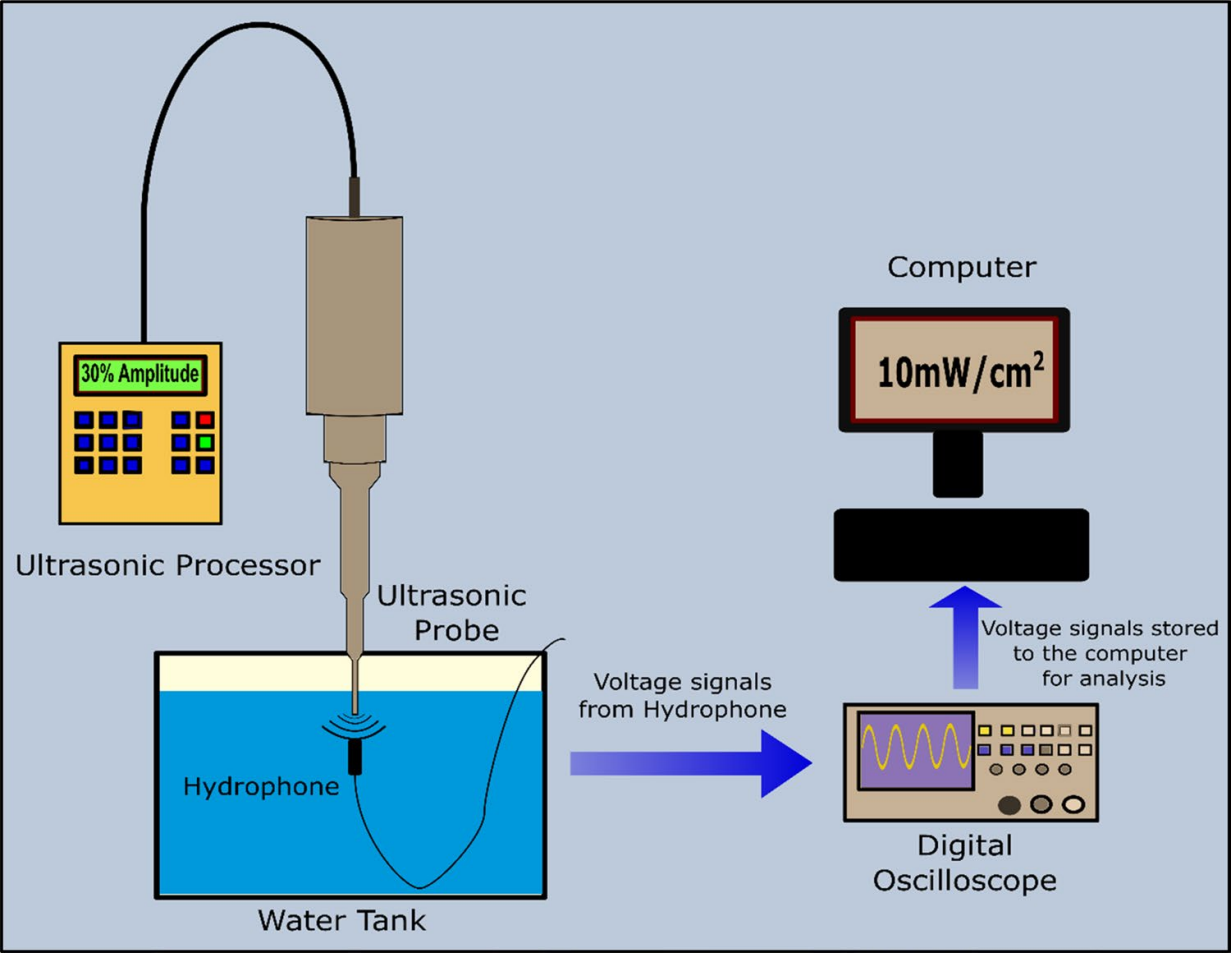
A schematic diagram showing the experimental setup for measuring power densities.

LFUS waves at 20-kHz were produced via an ultrasonic probe (model VCX750, Sonics & Materials Inc., Newtown, CT). The processor was used to generate three different amplitudes delivered to the probe, which are 20%, 25% and 30% as shown on the display of the machine. Accurate measurements of the acoustic power densities corresponding to these three amplitudes were carried out using a hydrophone (Bruel & Kjaer 8103, Nærum, Denmark). The probe was inserted in the water (2 cm from the surface of the water) and the hydrophone was placed in the water bath at a constant depth (3 cm from the tip of the probe). When US is applied, the hydrophone detects the pressure variations in the medium produced by the ultrasonic waves and converts these events into voltage signals. The signals are then fed to a digital storage oscilloscope (Tektronix TDS 2002B) and later analyzed using the MATLAB software (Fig. 3). While maintaining a constant distance in the ‘Z’ direction (the depth of the hydrophone), the hydrophone was raster scanned through an area of 7 cm × 5 cm (in X–Y direction) around the probe, and the signals were picked up at intervals of 1 cm. The measurements were repeated at the three investigated probe amplitudes mentioned-above. The measured voltage signals were then converted into acoustic pressure in Pascal using the equation:

1 $$P = \frac{Vrms\;(V)}{{Voltage \;Sensitivity\;\left( {\frac{\mu V}{{Pa}}} \right)}}$$

The value of the hydrophone voltage sensitivity was provided by the hydrophone manufacturer as 30 µV/Pa.

And the ultrasound power density ‘*I’* in Watt/cm^2^ is given by the equation

2 $$Power\;Density, \;\;I = \frac{{P^{2} }}{Z}$$

where ‘*Z*’ is the acoustic impedance of the medium (1.48 × 10^6^ kg m^−2^ S^−1^, the impedance of water) and ‘*P*’ is the pressure measured in Pascals.

### Low-frequency ultrasound release studies (Online Experiments)

**Figure 4:**
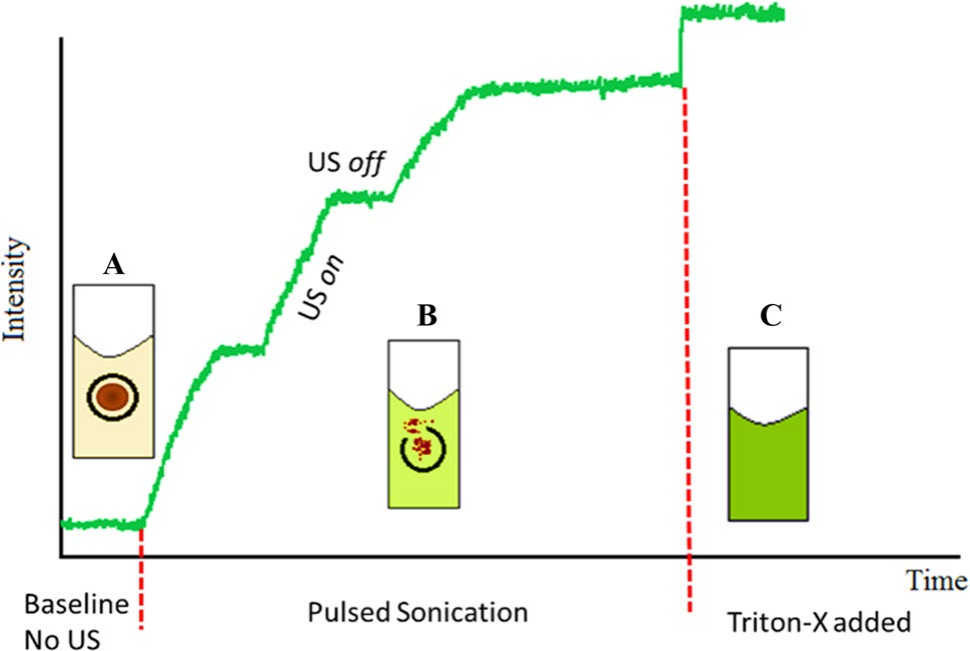
Calcein release from calcein-loaded liposomes following pulsed LFUS sonication. Calcein is self-quenched inside the liposomes showing low initial fluorescence level (**A**). The pulsed LFUS results in calcein release from the liposomes leading to the increase in calcein fluorescence (**B**). The addition of Tx-100 results in the maximum calcein release (**C**).

The release of calcein from liposomes was triggered using a 20-kHz low-frequency (LFUS) ultrasonic probe (model VCX750, Sonics & Materials Inc., Newtown, CT, USA) and monitored by fluorescence changes using a QuantaMaster QM 30 Phosphorescence Spectrofluorometer (Photon Technology International, Edison NJ, USA). Calcein is a fluorescent molecule with excitation and emission wavelengths of 495 and 515 nm, respectively. Samples were t prepared by diluting 75 μl of liposomes in 3 ml of PBS in a fluorescence cuvette^47^. The initial fluorescence intensity, *I*_0_, was measured for 60 s before applying US in a pulsed mode with 20 s *on* and 10 s *off*. The release was performed using three different power densities, 6.2 mW/cm^2^, 9 mW/cm^2^ and 10 mW/cm^2^. Three replicates were used for each sample. The pulsed mode was continued until a fluorescence plateau was reached, at which point 50 μl of Triton X-100 (Tx100) were added to the sample to lyse liposomes and release all the encapsulated calcein^47^. The recorded fluorescent intensities obtained experimentally were used to calculate the percentage of the released calcein (cumulative fraction released (CFR)) using the following equation^41,47^:

3 $$CFR = \frac{{I_{t} - I_{o} }}{{I_{\infty } - I_{o} }}$$

where *I*_0_ represents the baseline intensity, *I*_*t*_ represents the intensity at time *t*, and *I*_*∞*_ represents the highest fluorescence intensity value obtained. Figure 4 shows calcein release from the calcein-loaded liposomes following pulsed sonication using 20-kHz LFUS.

### Cell experiments

The HeLa cervical cell line was cultured in RPMI medium supplemented with 10% heat-inactivated fetal bovine serum (FBS) and 1% penicillin–streptomycin (Sigma-Aldrich, St. Louis, MO). The cultures were maintained at 37 °C in a humidified atmosphere with 5% CO_2_. Cells were routinely passaged every 2–3 days and split when reaching confluence. For the cellular uptake of the liposomes’ studies, exponentially growing cells were harvested with 3 ml of trypsin (0.25% from Sigma-Aldrich, St. Louis, MO) and 3 × 10^5^ cell/ml of growth media were seeded in 6-well plates to reach confluency at the time of the experiment^30^.

### Cell viability assay

Hela cells were seeded in a 6-well plate at a concentration of 6 × 10^5^ cells/well. Following overnight incubation, the media were replaced, followed by LFUS exposure for 5 min in a 35-kHz sonicating bath. Non-sonicated plates were used as a control. The cells were incubated for another 2 h and were then detached using Trypsin EDTA. Cell viability was determined by the Trypan Blue dye exclusion method.

### Flow cytometry analysis

Cells (3 × 10^5^ cells/mL) were cultivated in 6-well plates for 24 h. Both the control and Tf-PEG liposomes were added to the 6-well plates, followed by sonication using a 35-kHz US bath for 5 min. The sonicated plates were then incubated for 1 h at 37 °C in a humidified atmosphere with 5% CO_2_. Following the incubation period, each well was washed with PBS buffer and harvested using a trypsin solution. Harvested samples were then analyzed by measuring calcein fluorescence intensity using a flow cytometer [FC 500 (Beckman Coulter FC 500, US)]. In each insonation experiment, the sonolysis studies (the effect of US on cell viability) were performed using the Trypan blue exclusion assay. A minimum of three independent assays was performed for each treatment.

### Statistical analysis

Results were reported as average ± standard deviation (SD). A two-tailed *t *test was used to compare the sizes of the control and Tf-PEG liposomes, while two-factor ANOVA tests were employed to analyze LFUS and HFUS release results and kinetic modeling findings. Both types of ANOVA tests assumed that both populations have similar variances, and two values would be considered statistically different if p < 0.05 and if F < F_critical_ (unless otherwise stated).

## Results

### Physical properties and stability of the liposomes

**Figure 5:**
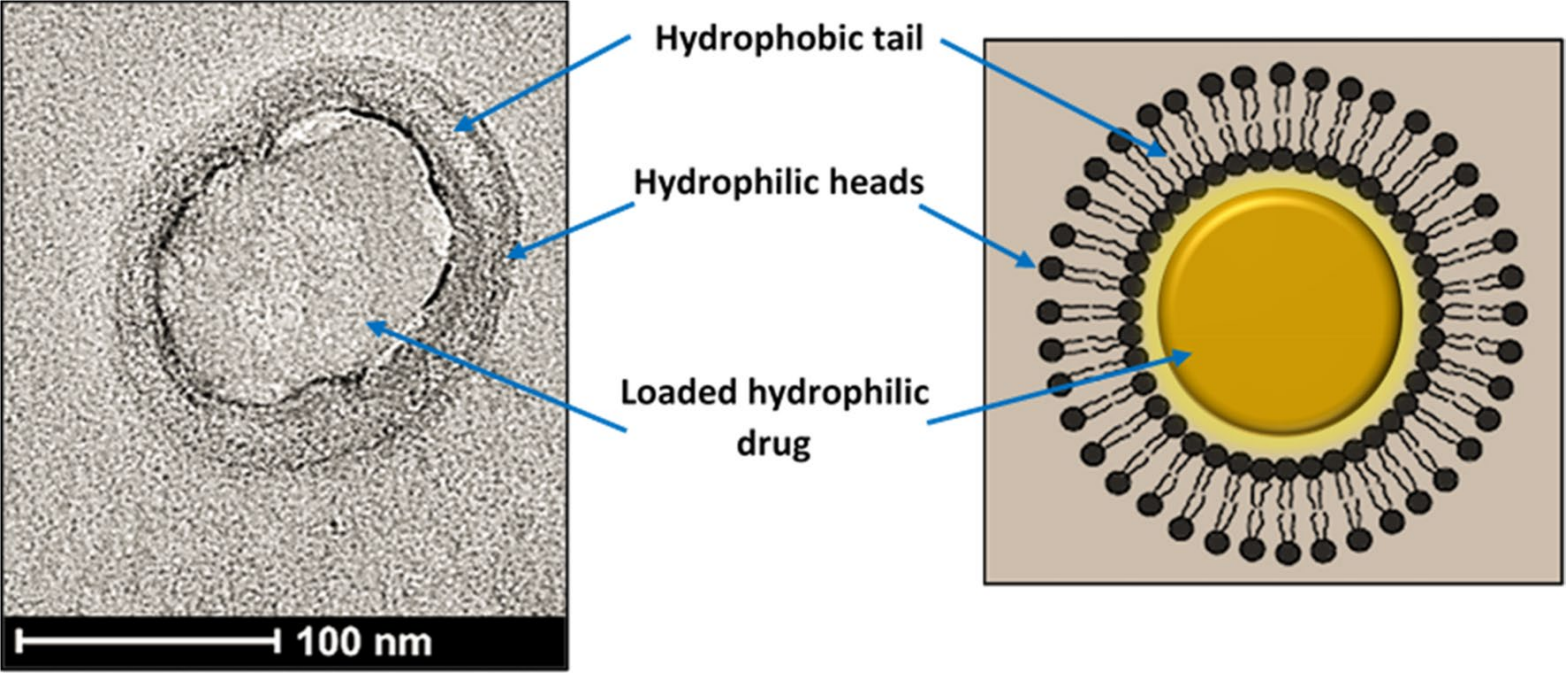
Transmission electron microscopy (TEM) images of calcein-loaded Tf-PEG liposomes at 100 nm scale and an illustration of the liposomes loaded with a hydrophilic drug.

The size of the prepared liposomes was measured using DLS. A polydispersity (Pd) index upper limit of 20% is generally acceptable for DLS measurements. Three replicates were used for each sample. The average diameter for the control liposomes was found to be 82.70 ± 2.88 nm, with a percent polydispersity index of 14.44 ± 1.72. The Tf-PEG liposomes were found to be slightly larger particles with an average diameter of 87.54 ± 4.81 nm and a Pd of 14.39 ± 2.42. Based on these findings, both types of liposomes fall within the size range of small lamellar vesicles (SLVs) and are well within the optimal size range for the EPR effect to take place. In addition, the results of the two-tailed *t* test analysis yielded a p-value of 0.0264, indicating that the two types had statistically different sizes. Figure 5 shows the transmission electron microscopy (TEM) image of calcein-loaded Tf-PEG liposomes together with an illustration of the liposomes loaded with a hydrophilic drug.

The Stewart assay confirmed that both the control and Tf-PEG liposomes had similar phospholipid concentrations. Tf-PEG liposomes showed a threefold increase in protein content compared to the control liposomes (0.646 ± 0.002 μg/mL and 1.98 ± 0.012 μg/mL respectively, p = 0.0174).

**Figure 6:**
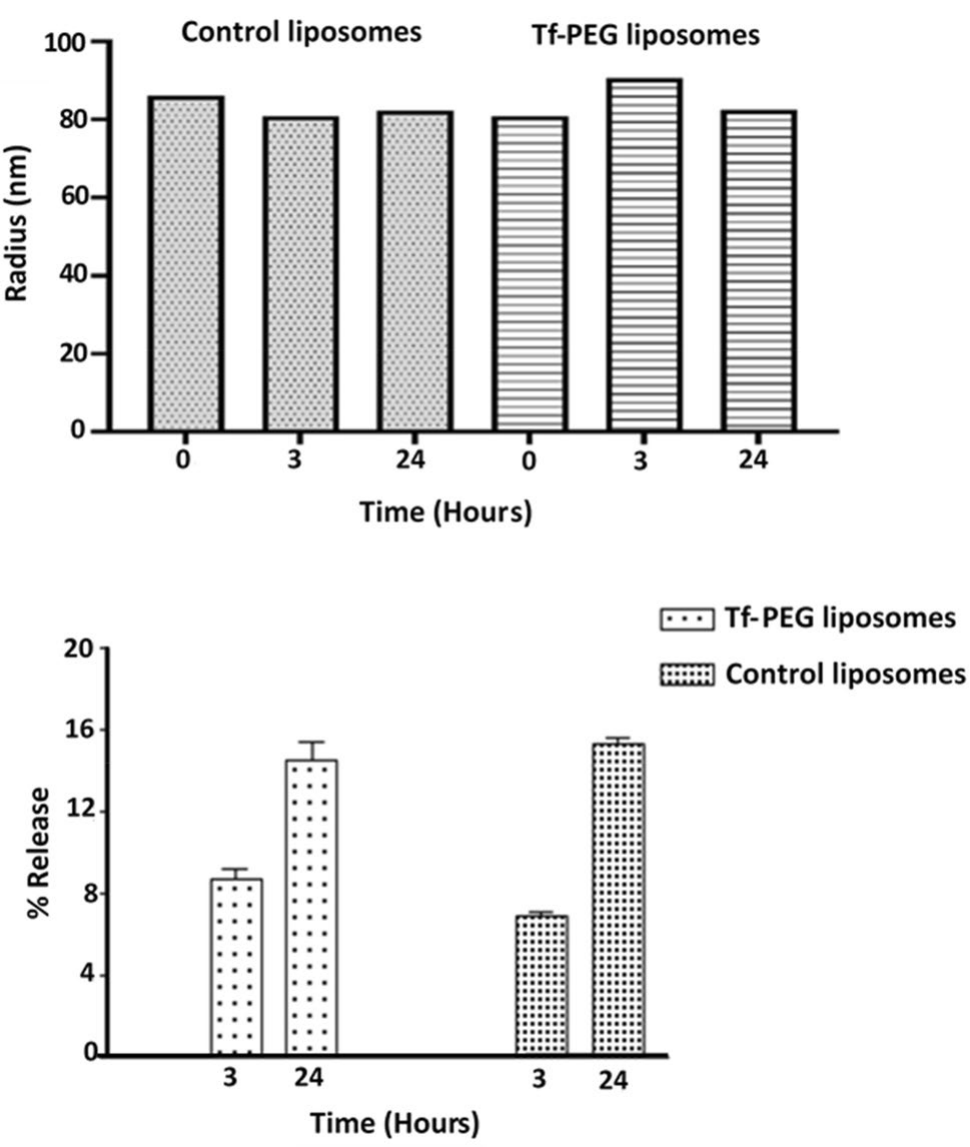
Average radius (top) and percentage calcein release (bottom) for the control and Tf-PEG liposomes following the incubation at 37 °C in fetal bovine serum medium for 3 h and 24 h. Results are the average of three liposome batches.

The stability of both types of liposomes was investigated to determine if these nanoparticles retain their structural integrity. This was achieved by incubating the liposomes at the biological temperature (i.e., 37 °C) in fetal bovine serum medium for 3 h and 24 h. Both the change in size and the release rate of the encapsulated calcein were recorded. As seen in Fig. 6, no significant change was observed in the control liposomes' size following the longest incubation period (i.e., 24 h), showing a radius of 82.3 ± 3.07 nm compared to 86.1 ± 1.98 nm before incubation (p-value = 0.152). Similarly, no significant difference in the Tf-PEG liposomes' size was observed, showing a size of 80.8 ± 0.75 nm and 82.4 ± 0.76 nm at 0 h and 24 h incubation, respectively (p-value = 0.057). This indicates that both types of liposomes are stable and maintained their structure after 24 h. The liposomes' ability to retain their cargo was investigated by measuring the release of calcein following the incubation period. As shown in Fig. 6, both types of liposomes released a small amount of the encapsulated calcein following 24 h of incubation, with release values of 16% and 15% for the control and Tf-PEG liposomes, respectively.

### Power density measurements

Accurate measurements of the three different power densities produced by the LFUS processor represented by percentage amplitudes of 20%, 25%, and 30% were measured using a hydrophone. The hydrophone was kept at a constant depth from the surface of the water while the distance from the tip of the sonicating probe was changed to produce a small 2D-acoustic map. The map was then used to determine the highest power density recorded at the three amplitudes tested here. The power densities produced by the processor were 6.2 mW/cm^2^ (20% amplitude), 9 mW/cm^2^ (25% amplitude) and 10 mW/cm^2^ (30% amplitude). Figure 1 (Supplementary Material) shows the measured power intensity values of an area at a distance of 3 cm from the probe's tip at amplitudes of 25% and 30%.

### Low-frequency ultrasound (LFUS) release

**Figure 7:**
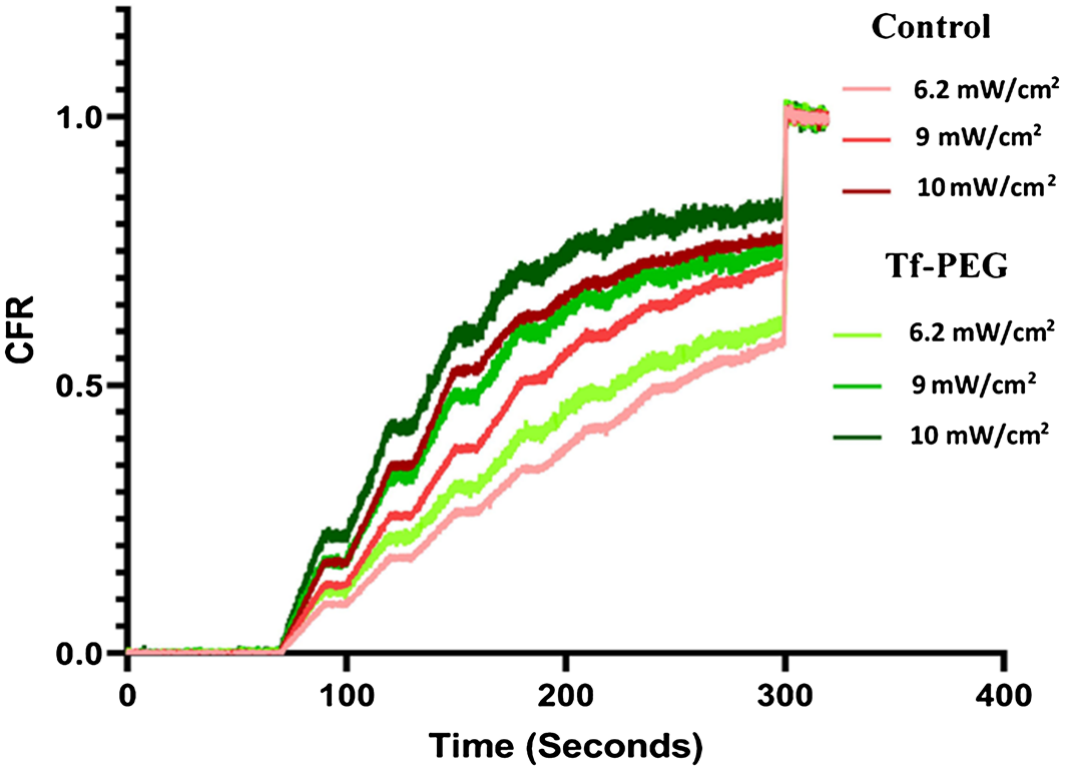
Normalized-averaged release profiles of 3 batches of control liposomes and Tf-PEG liposomes.

Low-frequency US release was measured for both the control and Tf-PEG liposomes using a minimum of three batches each (three replicates per batch) using a 20-kHz probe at three different power densities (6.2 mW/cm^2^, 9 mW/cm^2^ and 10 mW/cm^2^). The fluorescent intensity of the released calcein was measured using a QuantaMaster QM 30 Spectrofluorometer (Photon Technology International, Edison NJ, USA). The normalized-averaged release profile for the control and Tf-PEG liposomes are shown in Fig. 7. As can be seen, calcein encapsulated inside the liposome was at a self-quenched concentration of 1 mM showing little to no fluorescence reading and was used as the baseline. The application of pulsed LFUS triggered calcein release, thus, reducing the self-quenching of the model drug, and showing an increase in the fluorescence readings. Following 5 min of pulsed insonation, Tx-100 was added to lyse the liposomes, and the fluorescence measurements obtained were slightly above the preceding plateau, which indicated that the liposomes had released most of their encapsulated contents.

**Figure 8:**
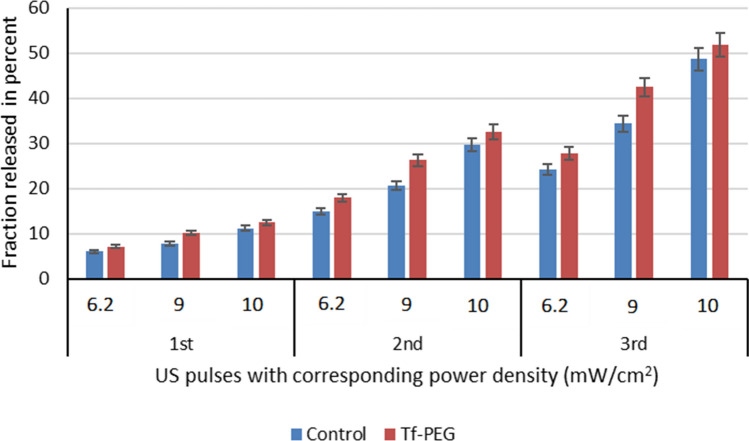
Percentage calcein release from both the control and Tf-PEG liposomes following the exposure to the first four pulses at the different power densities 6.2, 9 and 10 mW/cm^2^ expressed in terms of CFR.

 Figure 8 shows a comparison between the first three pulses at each power density in terms of CFR. The statistical analysis of the results showed that the percentage of release after the first pulse increased significantly (p < 0.05) as the power density was increased for both the control and Tf-PEG liposomes. The percentage release at the lowest power density used (6.2 mW/cm^2^) was significantly lower than that measured at 9 mW/cm^2^ with a p-value of 1.75 × 10^–8^ for the control, and 1.95 × 10^–11^ for the Tf-PEG liposomes. Similarly, when the release at 9 mW/cm^2^ was compared to that obtained at the highest power density of 10 mW/cm^2^, a significant difference was also observed (p-value of 9.74 × 10^–14^ and 7.53 × 10^–5^ for the control and Tf-PEG liposomes, respectively). A similar pattern was observed in percentage calcein release following the second and third pulses (p < 0.05) for both types of liposomes, where the release increased with the increase in power density.

A comparison between the control and Tf-PEG liposomes in terms of US-actuated calcein release is shown in Fig. 8. Overall, the statistical analysis showed that Tf-PEG liposomes exhibited higher fraction release when compared to the control liposomes at all three power densities investigated in our experiments (p < 0.05). The highest power density used (10 mW/cm^2^) is the most efficient in triggering calcein release from both types of liposomes.

We have also measured the size of the liposomes, using DLS, following the first three pulses of US to investigate the integrity of the liposomal membranes following exposure to acoustic power. As shown in Table 1, both the control and Tf-PEG liposomes maintained their integrity/structure and the polydispersity indices, for both types of liposomes, were within acceptable ranges following the 3rd and even the 6th pulses of US at all power densities investigated (6.2, 9 and 10 mW/cm^2^). Generally, the size of the liposomes got smaller as the US pulses increased to reach their smallest size following the 6th pulse. This indicates that sonicating the liposomes using LFUS triggered calcein release through transient pore formation rather than the total destruction of the liposomal structure.

**Table 1:** Size (radius) and polydispersity percentage (Pd %) of the control and Tf-PEG liposomes following the 3rd and 6th LFUS pulses. Results are the average of three batches.

	Before sonication	After 3rd pulse			After the 6th pulse		
		6.2 mW/cm^2^	9 mW/cm^2^	10 mW/cm2	6.2 mW/cm^2^	9 mW/cm^2^	10 mW/cm^2^
**Control**							
Radius (nm)	87.5 ± 2.57	73.7 ± 1.14	70.8 ± 0.424	70.7 ± 1.71	66.3 ± 2.18	62.1 ± 1.58	57.2 ± 1.23
*p*-value		0.226	0.000387	0.000417	0.000574	0.000147	5.02 × 10^–5^
Pd (%)	14.1 ± 2.95	16.6 ± 0.759	16.9 ± 1.21	15.3 ± 5.61	19.8 ± 2.90	21.8 ± 2.05	22.4 ± 1.05
*p*-value		0.198	0.233	0.210	0.0424	0.00768	0.0120
**Tf-PEG**							
Radius (nm)	80.5 ± 1.89	77.1 ± 1.25	76.4 ± 2.15	73.0 ± 1.25	74.1 ± 1.79	71.6 ± 2.04	69.7 ± 4.68
*p*-value		0.0366	0.0460	0.000579	0.000611	0.00136	0.01125
Pd (%)	9.1 ± 2.70	16.3 ± 3.88	18.6 ± 1.61	20.2 ± 2.10	21.3 ± 2.19	23.7 ± 0.063	23.9 ± 0.23
*p*-value		0.0364	0.00115	0.000637	0.000103	3.75 × 10^–5^	3.45 × 10^–5^

### Kinetic modeling of drug release

The real-time data collected using LFUS release were fitted to the linearized forms of the previously discussed models. The concentration or amount of drug released from the liposomes was represented by the cumulative fractional release (CFR), and the fit of each model equation was assessed in terms of the value of the coefficient of determination, R^2^.

For both types of liposomes, the best fitting model was the Korsmeyer–Peppas model. This model enables the estimation of the drug release mechanism using the exponential value *n*. The value of n = 0.8135 for control liposomes and 0.7207 for Tf-PEG liposomes. Based on the range of *n* values detailed in Table 2, the diffusional release mechanism governing both control and Tf-PEG liposomes is anomalous transport, i.e., the diffusion process has a non-linear relationship with time.

**Table 2:** Diffusional drug release from polymeric systems^49^.

Release exponent (n)			Drug transport mechanism	Rate as a function of time
Cylinder	Sphere	Thin film		
0.5	0.43	0.5	Fickian diffusion	t^−0.5^
0.45 < n < 0.89	0.43 < n < 0.85	0.5 < n < 1	Anomalous transport	t^n−1^
0.89	0.85	1	Polymer swelling	Zero-order release

The values of release constants obtained from the linearized model equation were compared using a two-way analysis of variance (ANOVA), the findings are summarized in Table 3. Comparing control and Tf-PEG liposomes, we note that the F-value is much higher than the critical F-value, and the p-value is much lower than alpha (standard α = 0.05). This suggests that there is enough evidence to reject the null hypothesis; meaning that the k-values are affected by the carrier type. Comparing release at different power densities, the F-value is also higher than the critical F-value, and the p-value is also much lower than 0.05. Thus, changes in power density have significant effects on k-values. This difference may be due to the impact of the conjugated moieties on reducing liposomes' stability, which significantly affects the release kinetics (causing the rate constants to increase with increased power density). Finally, the interaction p-value is greater than 0.05, indicating no interaction between the two independent factors. Although the prediction of the release constant values by this model was not very accurate, its applicability still holds due to the perfect fitting (high R^2^-values) demonstrated earlier^47^.

**Table 3:** Two-way ANOVA test for release constant values of Korsmeyer–Peppas model.

ANOVA Source of variation	SS	df	MS	F	p-value	F-crit
Sample	0.000244	1	0.000244	16.48807	0.00158	4.747225
Columns	0.000397	2	0.000199	13.42287	0.000869	3.885294
Interaction	1.36E−05	2	6.81E−06	0.460363	0.64175	3.885294
Within	0.000178	12	1.48E−05			
Total	0.000833	17				

### Effect of LFUS on cell viability

HeLa cells sonicated using LFUS (35-kHz) showed no significant difference in cell viability compared to the non-sonicated control cells (Table 4). This shows that US caused no significant destruction/killing of the cancer cells (p-value > 0.05), and hence can be used as a drug triggering modality.

**Table 4:** Cell viability following a 5-min sonication with LFUS (35-kHz).

	Control	Sonicated	p-value
Viability %	96.9%	97.3%	0.845
Std.Dev	2.45	2.99	

### Cellular uptake of Tf-conjugated calcein liposomes vs. non-targeted liposomes

**Figure 9:**
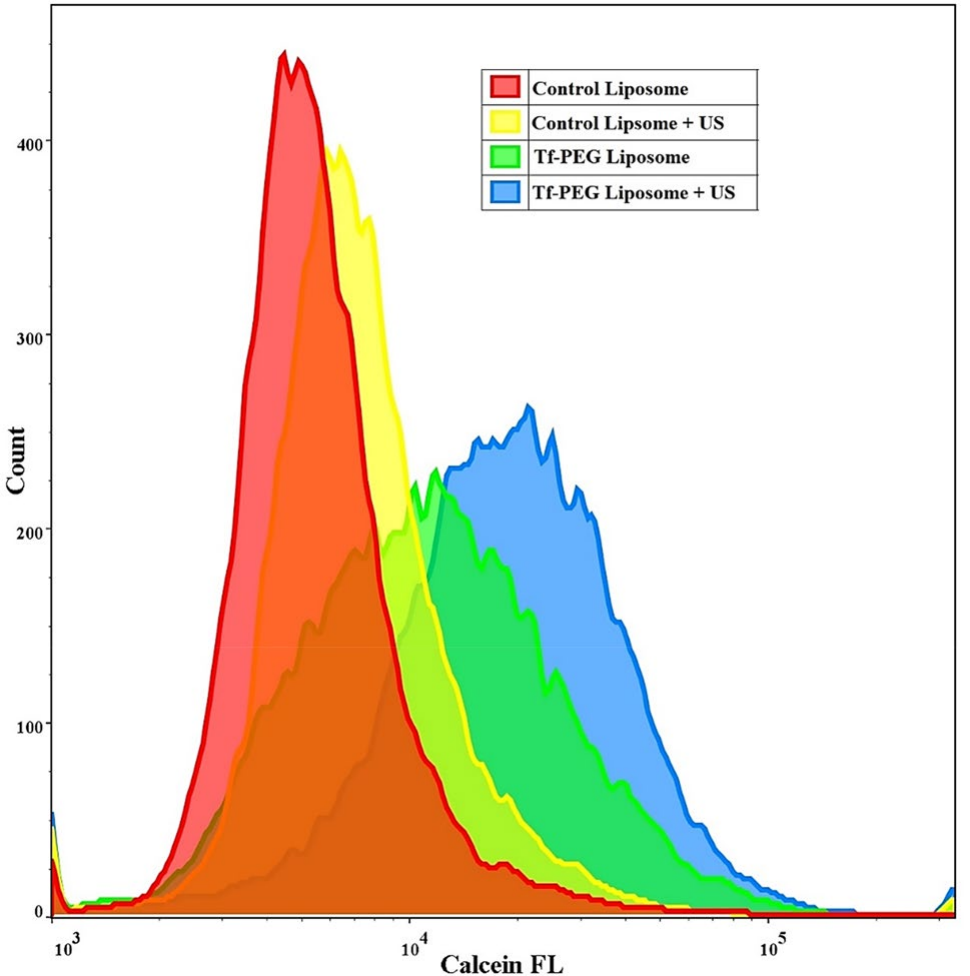
Calcein uptake by HeLa cells incubated with control and Tf-PEG liposomes and exposed to LFUS (35-kHz). Results are the average ± standard deviation of three liposome batches (3 replicates each).

A comparison between the geometric means of the subcellular internalization of calcein by HeLa cells using flow cytometry was conducted. Figure 9 shows that, on average, calcein uptake from Tf-PEG liposomes was 74% more than calcein uptake from the control liposomes (p-value = 0.008). The continuous need for iron supply results in the overexpression of Tf receptors by HeLa cells. Generally, HeLa cells expressed 2.0 × 10^5^ receptors/cell^50^, and following Tf binding to the TfR1 receptor, HeLa cells uptake of Tf is completed through receptor-mediated endocytosis using clathrin-mediated endocytosis^51,52^. Therefore, Tf conjugation to the liposomes enhanced liposomal uptake by HeLa cells through the clathrin-mediated endocytosis route^53,54^. Furthermore, the exposure of HeLa cells incubated with control liposomes to LFUS (at 35-kHz and 1 W/cm^2^) increased the average calcein uptake by 18% compared to the uptake without sonication (p-value = 0.002). Similarly, Tf-PEG liposomes incubated with HeLa cells showed a higher level of cellular uptake following acoustic exposure, with an average increase of 42% (p-value = 0.01). Thus, there is a 151% increase in calcein uptake by the sonicated cell compared to the uptake from the non-sonicated control liposomes (p-value = 0.0004).

## Discussion

Chemotherapy is an effective and successful method to treat many types of cancer. However, the common side effects associated with this type of treatment usually reduce the quality of life of cancer patients and affects them physically and mentally. This study is part of the ongoing research aiming to reduce the toxicity and enhance the efficiency of chemotherapeutic drugs by reducing their contact with the healthy cells in the body. This is achieved by encapsulating anti-neoplastic agents inside nano-sized liposomes designed to target specific receptors overexpressed on the surfaces of the cancer cells. We have synthesized pegylated liposomes encapsulating calcein and decorated their surfaces with transferrin molecules (Tf-PEG liposomes) to target the overexpressed Tf receptors present on the surface of the cervical cancer cell line (the HeLa cell line) to supply the high demand for iron. Following the binding of Tf-PEG liposomes to the receptors, low-frequency ultrasound (LFUS) was applied to enhance the release of the encapsulated calcein in a spatiotemporally controlled manner. While the LFUS sonicating probe was used in this study to show that LFUS triggered calcein release from different liposomal formulations, all other experiments involving cells were conducted using a 35-kHz sonicating bath.

**Figure 10:**
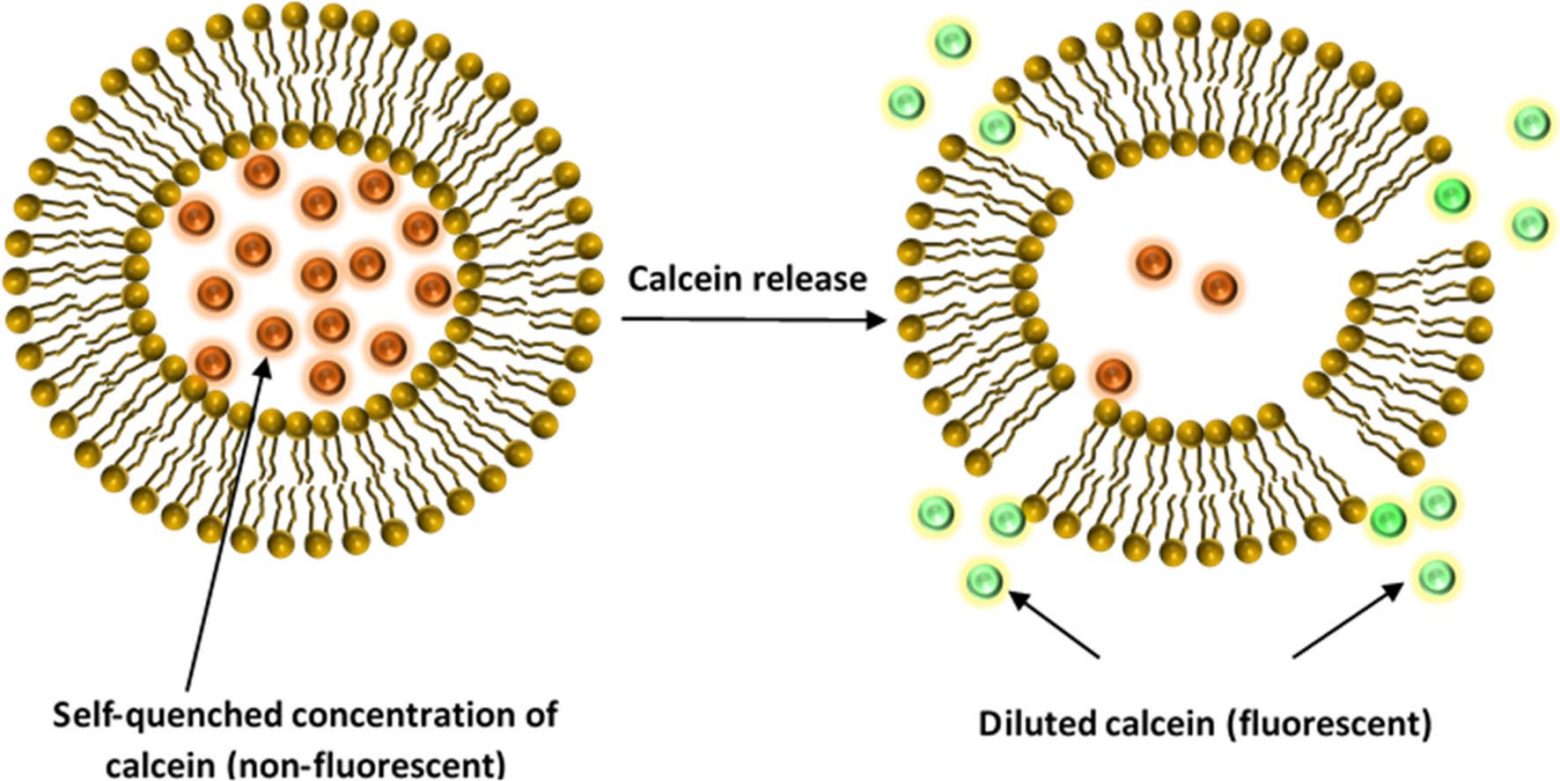
Schematic representation of liposomes encapsulating calcein at a self-quenched concentration. Calcein becomes fluorescent when released and diluted in the aqueous surroundings.

Calcein is a self-quenching fluorescent dye. This phenomenon is utilized to investigate the cellular uptake of both liposomes and the fluorescent dye/model drug. Calcein is encapsulated in the inner compartment of the liposomes at a high (self-quenched) concentration. Thus, calcein exhibits very low fluorescence inside the liposomes. High levels of fluorescence, measured via flow cytometry, are due to the release (leakage) of calcein from liposomes and the subsequent dilution in the exterior aqueous surroundings. This occurs when liposomes are taken up by the cells and their contents released, which is then diluted in the cytoplasm allowing its fluorescence to be detected at a higher level (Fig. 10).

Therefore, the flow cytometry detection of calcein inside the cells is correlated to the amount of liposomes taken up by the cells. Low calcein fluorescence inside the cells indicates that a small amount of liposomes and dye are present inside and vice versa. Calcein encapsulation inside the liposomes was already shown to improve calcein uptake by the cancer cells^55^. In our present study, we compared the uptake of calcein encapsulated inside conventional liposomes (non-targeted) compared to targeted liposomes (Tf-liposomes) with and without US triggering. It is expected that targeted liposomes will be taken up more by the cells compared to the non-targeted liposomes due to the added benefit of binding and receptor-mediated endocytosis. This means that more of the encapsulated calcein is taken up by the cells and thus, higher calcein fluorescence intensity will be detected inside the cells treated with targeted liposomes compared to those treated with non-targeted liposomes as was shown in previous studies^56–59^. Sonication with LFUS will trigger calcein release from the liposomes by creating transient pores in the liposomes’ phospholipid bilayer. Also, the sonoporation effect on the cellular membranes allows higher uptake of the liposomes. The third mechanism at play entails liposomes releasing their contents outside the cells and the drug diffusing through their cell membrane due to sonoporation. Therefore, it is expected that US triggering will further increase the amount of calcein detected inside the cells due to these three mechanisms.

The size of the synthesized control and Tf-PEG liposomes was within the recommended size of nanoparticles used in drug delivery (< 200 nm). This allows the liposomes to extravasate through the pores formed in the leaky tumor vasculature, also known as the enhanced permeability and retention effect (EPR). Both types of liposomes showed good percentages of polydispersity and showed similar stability when incubated at the biological temperature (i.e., 37 °C) for 24 h in terms of maintaining their size and retaining the loaded calcein. We investigated the effect of pulsed LFUS (20 s on, 10 s off) in triggering the release from both the control and Tf-PEG liposomes. While the frequency and pulse duration were kept constant, calcein release, using three different power densities, was compared. Generally, calcein release increased with the increase in power density and the release was detected during the “on” pulse period but froze during the “off” period. This indicates that LFUS was the main trigger of calcein release rather than other factors such as lipid oxidation.

Previous studies have also shown that US triggers drug release from liposomes in a controlled manner^28,37,60^. Both the mechanical and thermal acoustic effects could play a role in triggering calcein release from the liposomes. The highest temperature recorded following the third pulse for all the power densities used was 31 °C, which is significantly lower than the transition temperature of the main phospholipid used to prepare the liposomes (i.e., DPPC) of 41.3 °C. Although the thermal effect could still play a role in triggering calcein release, cavitation is likely to be the main culprit behind this release phenomenon. The mechanical index (MI) is a parameter used to indicate the possibility of the occurrence of cavitation and is mathematically represented by Eq. (4).

4 $$MI = \frac{{P_{neg} }}{\sqrt f }$$

The negative pressure (expressed in units of MPa) in the above equation is dependent upon the acoustic impedance of water, Z, (for human soft tissues, the values of Z are comparable to those of water), and the intensity of the LFUS. Mathematically, negative pressure is denoted by Eq. (5) below^61^.

5 $$P_{neg} = \sqrt {2 Z I}$$

For water, the acoustic impedance has a value of 1.48 MPa s/m^62,63^, and for the LFUS power densities used in this research, namely 6.2, 9 and 10 mW/cm^2^, the calculated MI values are 0.11, 0.12, and 0.16, respectively. The threshold of collapse cavitation is expected to occur at around MI = 0.3, biological effects are observed at MI > 0.7, and tissue damage is expected to occur at MI > 1.9^42,61,64,65^. Therefore, the power densities used in this work are well below the collapse cavitation threshold of 0.3, thus implicating stable cavitation as the primary cause of the observed release.

Liposomes have a similar structure to cellular membranes. The cavitation produced by US waves is known to increase the permeability of biological membranes^66^. Earlier studies have shown that cavitation-mediated drug release from liposomes is mainly achieved by increasing their permeability through the formation of pores in their phospholipid membranes^35,36^. Pegylation was also reported to enhance their permeability following sonication with LFUS compared to non-pegylated liposomes^28,37^. The lower packing parameter of DSPE-PEG, compared to other phospholipids like DPPC^67^, could result in ejecting the DSPE-PEG out of the phospholipid membrane when exposed to LFUS waves, thus enhancing membrane permeability. The higher calcein release from Tf-PEG liposomes compared to the control pegylated liposomes reported here could be due to the increase in DSPE-PEG weight following Tf conjugation. Tf has a molecular weight of 80 kDa. This extra weight increases the possibility of DSPE-PEG ejection from the liposomes resulting in increasing the permeability and, subsequently, an increase in drug release from the liposomes. Interestingly, we found that following the first three pulses of LFUS, the size of both the control and Tf-PEG liposomes got smaller at all the power densities used in this work. We also measured the size after 6 pulses of LFUS and found that the size of both types of liposomes continued to get smaller . This is in agreement with previous studies that reported the decrease in liposome size following exposure to US^29,68^. This could be because liposomes undergo transient changes due to pore formation in their phospholipid wall, small fractions of the lipids are forced out and as the wall re-seal, the size of the liposomes gets smaller.

We showed that exposing the cells to LFUS (35-kHz) showed no cytotoxicity as no significant difference in the viability of HeLa cells sonicated with LFUS compared to the non-sonicated cells was observed. HeLa cells were also incubated with the liposomes to test cellular uptake of both the control and Tf-PEG liposomes with and without sonication with LFUS. Calcein is a fluorescent probe and can be detected using flow cytometry to measure the amount of this model drug present inside the cells. When cells are incubated with control (non-targeted) liposomes encapsulating calcein, we expect calcein to be detected inside the cells as the cells associate with the nanocarriers through simple adsorption because of the surface interaction between the liposomes and the cell through electrostatic forces. This was represented by the first red peak. When US was applied, sonication resulted in the formation of transient pores in the walls of the control liposomes as well as in the cellular membrane of the HeLa cells. This resulted in increasing the amount of calcein inside the cells which was reflected by the shift from the red peak to the yellow peak, as shown in Fig. 9. This is in agreement with previous studies that showed US exposure to increase the cellular uptake of liposomes^69,70^.

Conjugating targeting moieties to the surface of the liposomes such as those used in this study (Tf conjugated liposomes or Tf-PEG), will result in the binding of those liposomes to Tf receptors overexpressed on the surface of the HeLa cells. This will allow more liposomes to be taken up by the cells compared to the control liposomes; thus, more calcein was detected inside the cells, shifting the peak from red to green (Fig. 9). The increase in calcein uptake is not due to the reduced stability of the Tf-PEG liposomes compared to the control liposomes, as we showed in Fig. 6, where both liposomes showed similar stability. Therefore, the higher calcein uptake from the Tf-PEG liposomes compared to the control liposomes is mainly due to the enhanced cellular uptake of the targeted liposomes. Since more targeted liposomes are already binding or taken by the cancer cells, US application will further enhance the amount of calcein present inside the cells, as represented by the final blue peak in Fig. 9.

## Conclusion

This study shows that combining transferrin conjugated liposomes (Tf-PEG liposomes) and low-frequency ultrasound is a promising technique in targeting cervical cancer . Future work should include in vivo experiments to determine the ultimate therapeutic efficacy of this drug delivery platform. Additionally, the optimization of US parameters, including the acoustic frequency, power density, and pulse duration, to enhance drug release and cellular uptake in vivo is underway.

## Supplementary Information

Supplementary Information.
