# Supplementary material for: Transferrin-modified liposomes triggered with ultrasound to treat HeLa cells
Source: Sci Rep. 2021 Jun 2;11:11589. doi: 10.1038/s41598-021-90349-6 (PMC8172941; doi:10.1038/s41598-021-90349-6)
Supplement: Supplementary file 1 — Supplementary Information. [file 41598_2021_90349_MOESM1_ESM.docx]

**Transferrin-modified liposomes triggered with ultrasound to treat HeLa cells**

Nour M. AlSawaftah^a^, Nahid S. Awad*^a^*, Vinod Paul*^a^*, Paul S. Kawak*^a^*, Mohammad H. Al-Sayah*^b^*, Ghaleb A. Husseini *^,^ *^a^*

*^a^Department of Chemical Engineering, American University of Sharjah, PO. Box 26666, Sharjah, UAE*

*^b^Department of Biology, Chemistry and Environmental Sciences, American University of Sharjah, PO. Box 26666, Sharjah. UAE*

E-mail: [ghusseini@aus.edu](mailto:ghusseini@aus.edu)

# Supplementary information:

# Model Derivation

# Zero Order Model.

The zero-order model can be used to describe the dissolution of drugs encased in non-disintegrating dosage forms, con­sidering a very slow drug release (with no changes in the equilibrium conditions) [1]–[5]. If the function *Q(t)* is assumed to represent the amount of the drug that is in solution at any time *t* then according to the zero order assumptions the behavior of the drug in solution is represented by:

|  | $\frac{\boldsymbol{dQ(t)}}{\boldsymbol{dt}}\boldsymbol{=}\boldsymbol{K}_{\boldsymbol{0}}$ | (1) |
| --- | --- | --- |

Where *K_0_* is a zero-order release constant which has units of concentration per unit time. Integrating with respect to time over the interval [0,t]:

|  | $\int_{\boldsymbol{Q}_{\boldsymbol{0}}}^{\boldsymbol{Q}_{\boldsymbol{t}}} \boldsymbol{dQ(t)}\boldsymbol{=}\int_{\boldsymbol{0}}^{\boldsymbol{t}} \boldsymbol{K}_{\boldsymbol{0}}\boldsymbol{dt}$ | (2) |  |
| --- | --- | --- | --- |
|  | $\boldsymbol{Q}_{\boldsymbol{t}}\boldsymbol{=}\boldsymbol{Q}_{\boldsymbol{0}}\boldsymbol{+}\boldsymbol{K}_{\boldsymbol{0}}\boldsymbol{t}$ | | (3) |

Re-defining the model in terms of the Cumulative Fraction Released (CFR):

|  | $\boldsymbol{CFR=}\frac{\boldsymbol{Drug released at any time, t-Initial Amount}}{\boldsymbol{Total amount of drug present}}\boldsymbol{=}\frac{\boldsymbol{Q}_{\boldsymbol{t}}\boldsymbol{-}\boldsymbol{Q}_{\boldsymbol{0}}}{\boldsymbol{Q}_{\boldsymbol{T}}}$ | (4) |
| --- | --- | --- |

Assuming that *Q_0_* is very small and can be presumed to be 0. Dividing equation (4) by the total amount of the drug released/present, *Q_T_*:

|  | $\boldsymbol{CFR=}\frac{\boldsymbol{K}_{\boldsymbol{0}}}{\boldsymbol{Q}_{\boldsymbol{T}}}\boldsymbol{t}$ | (5) |
| --- | --- | --- |

Introducing a constant *k_0_:*

|  | $\boldsymbol{k}_{\boldsymbol{0}}\boldsymbol{=}\frac{\boldsymbol{K}_{\boldsymbol{0}}}{\boldsymbol{Q}_{\boldsymbol{T}}}$ | (6) |  |
| --- | --- | --- | --- |
|  | $\boldsymbol{CFR=}\boldsymbol{k}_{\boldsymbol{0}}\boldsymbol{t}$ | | (7) |

The plot of CFR vs. time should give a straight line with a slope of *k_0_* passing through the origin.

# Hixson-Crowell.

Hixson and Crowell discovered that a particle’s area is propor­tional to the cube root of its volume. This equation applies to dosage forms in which the dissolution happens in planes parallel to the surface of the dosage form; this surface decreases proportionally over time but the geometrical form remains constant [1]–[3].

Starting with the Noyes-Whitney equation to describe the dissolution of the drug,

|  | $\frac{\boldsymbol{dW}}{\boldsymbol{dT}}\boldsymbol{=}\frac{\boldsymbol{kA(}\boldsymbol{C}_{\boldsymbol{s}}\boldsymbol{-}\boldsymbol{C}_{\boldsymbol{\infty}}\boldsymbol{)}}{\boldsymbol{l}}$ | (8) |
| --- | --- | --- |

Where *dW/dT* represents the rate of dissolution, *k* is the Noyes-Whitney constant and contains the diffusion constant *D*, *A* is the surface area, *C_s_* is the solute concentration, *C_o_* is the concentration of the bulk solution, which in this case is assumed to be zero, and *L* is the diffusion layer. Simplifying the previous equation gives:

|  | $\boldsymbol{dW=kA}\frac{\left( \boldsymbol{C}_{\boldsymbol{s}} \right)}{\boldsymbol{l}}\boldsymbol{dt}$ | (9) |
| --- | --- | --- |

The mass is represented as follows and the negative sign represents drug loss,

|  | $\boldsymbol{dW=-\rho dV}$ | (10) |
| --- | --- | --- |

Since liposomes are considered to be spherical in shape (*N* represents the number of spheres),

|  | $\boldsymbol{V=}\frac{\boldsymbol{4}}{\boldsymbol{3}}\boldsymbol{\pi}\boldsymbol{r}^{\boldsymbol{3}}\boldsymbol{N}$ | (11) |  |
| --- | --- | --- | --- |
|  | $\frac{\boldsymbol{dV}}{\boldsymbol{dr}}\boldsymbol{=4}\boldsymbol{\pi N}\boldsymbol{r}^{\boldsymbol{2}}$ | (12) | |

Next, combining equations 9 and 10 and substituting for *dV* and *A*, we get the following:

|  | $\boldsymbol{dW=-\rho}\boldsymbol{4}\boldsymbol{\pi N}\boldsymbol{r}^{\boldsymbol{2}}\boldsymbol{dr=}\frac{\boldsymbol{k}\boldsymbol{4}\boldsymbol{\pi N}\boldsymbol{r}^{\boldsymbol{2}}\boldsymbol{C}_{\boldsymbol{s}}\boldsymbol{dt}}{\boldsymbol{l}}$ | (13) |
| --- | --- | --- |

Integrating on both sides,

|  | $\int_{\boldsymbol{r}_{\boldsymbol{o}}}^{\boldsymbol{r}_{\boldsymbol{t}}} \boldsymbol{-\rho dr}\boldsymbol{=}\frac{\boldsymbol{k}\boldsymbol{C}_{\boldsymbol{s}}}{\boldsymbol{l}}\int_{\boldsymbol{0}}^{\boldsymbol{t}} \boldsymbol{dt}$ | (14) |  |
| --- | --- | --- | --- |
|  | $\boldsymbol{-}\left( \boldsymbol{r-}\boldsymbol{r}_{\boldsymbol{o}} \right)\boldsymbol{=}\frac{\boldsymbol{k}\boldsymbol{C}_{\boldsymbol{S}}\boldsymbol{T}}{\boldsymbol{\rho l}}$ | (15) | |
|  | $\boldsymbol{r}_{\boldsymbol{t}}\boldsymbol{=-}\frac{\boldsymbol{k}\boldsymbol{C}_{\boldsymbol{S}}\boldsymbol{T}}{\boldsymbol{\rho l}}\boldsymbol{+}\boldsymbol{r}_{\boldsymbol{o}}$ | (16) | |

To adapt the expression in terms of CFR, the weight of dissolution *W* is used instead of the volume. Therefore, for *N* spheres the volume is:

|  | $V=\frac{4}{3}\pi r^{3}N$ | (17) |
| --- | --- | --- |

The weight of each sphere can be expressed as:

|  | $\boldsymbol{W=\rho}\frac{\boldsymbol{4}}{\boldsymbol{3}}\boldsymbol{\pi}\boldsymbol{r}^{\boldsymbol{3}}\boldsymbol{N}$ | (18) |
| --- | --- | --- |

Raising all variables to the cubic root and re-arranging for *r*:

|  | $\boldsymbol{r=}\frac{\boldsymbol{W}^{\frac{\boldsymbol{1}}{\boldsymbol{3}}}}{\left( \boldsymbol{\rho}\frac{\boldsymbol{4}}{\boldsymbol{3}}\boldsymbol{\pi N} \right)^{\frac{\boldsymbol{1}}{\boldsymbol{3}}}}$ | (19) |
| --- | --- | --- |

Substituting equation 19 into equation 16 gives:

|  | $\frac{{\boldsymbol{W}_{\boldsymbol{t}}}^{\frac{\boldsymbol{1}}{\boldsymbol{3}}}}{\left( \boldsymbol{\rho}\frac{\boldsymbol{4}}{\boldsymbol{3}}\boldsymbol{\pi N} \right)^{\frac{\boldsymbol{1}}{\boldsymbol{3}}}}\boldsymbol{=-}\frac{\boldsymbol{k}\boldsymbol{C}_{\boldsymbol{S}}\boldsymbol{T}}{\boldsymbol{\rho l}}\boldsymbol{+}\frac{{\boldsymbol{W}_{\boldsymbol{o}}}^{\frac{\boldsymbol{1}}{\boldsymbol{3}}}}{\left( \boldsymbol{\rho}\frac{\boldsymbol{4}}{\boldsymbol{3}}\boldsymbol{\pi N} \right)^{\frac{\boldsymbol{1}}{\boldsymbol{3}}}}$ | (20) |
| --- | --- | --- |

Simplifying the above equation into the Hixson-Crowell form,

|  | ${\boldsymbol{W}_{\boldsymbol{t}}}^{\frac{\boldsymbol{1}}{\boldsymbol{3}}}\boldsymbol{=-}\frac{\boldsymbol{k}\boldsymbol{C}_{\boldsymbol{S}}\boldsymbol{T}}{\boldsymbol{\rho l}}\left( \boldsymbol{\rho}\frac{\boldsymbol{4}}{\boldsymbol{3}}\boldsymbol{\pi N} \right)^{\frac{\boldsymbol{1}}{\boldsymbol{3}}}\boldsymbol{+}{\boldsymbol{W}_{\boldsymbol{o}}}^{\frac{\boldsymbol{1}}{\boldsymbol{3}}}$ | (21) |
| --- | --- | --- |

Let *K’* represent the constant related to the surface, the shape and the density of the particle,

|  | ${\boldsymbol{W}_{\boldsymbol{0}}}^{\frac{\boldsymbol{1}}{\boldsymbol{3}}}{\boldsymbol{-W}_{\boldsymbol{t}}}^{\frac{\boldsymbol{1}}{\boldsymbol{3}}}\boldsymbol{=}\frac{\boldsymbol{k}\boldsymbol{K}^{\boldsymbol{'}}\boldsymbol{C}_{\boldsymbol{S}}\boldsymbol{T}}{\boldsymbol{l}}\left( \boldsymbol{N} \right)^{\frac{\boldsymbol{1}}{\boldsymbol{3}}}$ | (22) |
| --- | --- | --- |

Re-defining the model in terms of CFR,

|  | $\boldsymbol{CFR=}\frac{\boldsymbol{Drug released at any time, t-Initial Amount}}{\boldsymbol{Total amount of drug present}}\boldsymbol{=}\frac{\boldsymbol{C}_{\boldsymbol{t}}\boldsymbol{-}\boldsymbol{C}_{\boldsymbol{0}}}{\boldsymbol{C}_{\boldsymbol{T}}}\boldsymbol{=}\frac{\boldsymbol{W}_{\boldsymbol{t}}\boldsymbol{-}\boldsymbol{W}_{\boldsymbol{0}}}{\boldsymbol{W}_{\boldsymbol{0}}}$ | (23) |
| --- | --- | --- |

Simplifying and linearizing the above equation yields:

|  | ${\boldsymbol{[}\left( \boldsymbol{1-CFR} \right)\boldsymbol{]}}^{\frac{\boldsymbol{1}}{\boldsymbol{3}}}\boldsymbol{=}\boldsymbol{K}_{\boldsymbol{HC}}\boldsymbol{t}$ | (24) |
| --- | --- | --- |

Here *K_HC_* represents the release constant and according to equation 81 a plot of *[(1-CFR)]^1/3^* versus time would yield a straight line with a slope of *K_HC_*.

# Korsmeyer-Peppas (Power Law) Model.

The Korsmeyer-Peppas model is a semi-empirical model, establishing the exponential relationship between the release and the time [1]–[4], [6], [7]:

|  | $\frac{\boldsymbol{M}_{\boldsymbol{t}}}{\boldsymbol{M}_{\boldsymbol{\infty}}}\boldsymbol{=K}\boldsymbol{t}^{\boldsymbol{n}}$ | (25) |
| --- | --- | --- |

*M*∞ is the amount of drug at the equilibrium state (sometimes very close to the amount of drug contained in the dosage form at the beginning of the release process), *M_t_* is the amount of drug released over time *t*, *K* is the constant of incorporation of structural modifications and geometrical characteris­tics of the system (also considered the release velocity constant), and *n* is the exponent of release (related to the drug release mechanism) in function of time *t*. In terms of CFR,

|  | $CFR=\frac{\boldsymbol{M}_{\boldsymbol{t}}}{\boldsymbol{M}_{\boldsymbol{\infty}}}\boldsymbol{=K}\boldsymbol{t}^{\boldsymbol{n}}$ | (26) |
| --- | --- | --- |

Taking the logarithms of both sides of the equation,

|  | $\log\left( \boldsymbol{CFR} \right)\boldsymbol{=}\log\left( K \right)\boldsymbol{+nlog}\left( \boldsymbol{t} \right)$ | (27) |
| --- | --- | --- |

Thus, if *log(CFR)* is plotted against the logarithm of time, a straight-line graph can be obtained with a y-intercept at *log(K),* and the slope is *n*.

Depending on the value of *n,* it is possible to establish a classification of the release mechanism, according to the type of observed behavior:

– Fickian model (Case I)

– Non-Fickian models (Case II, Anomalous Case and Super Case II)

In the Fickian model (Case I), the drug release is governed by dif­fusion. While if the model is non-Fickian, for Case II transport, the drug release rate corresponds to zero-order release kinetics and the mechanism driving the drug release is the swell­ing or relaxation of polymeric chains. For anomalous transport, the mechanism of drug release is governed by diffusion and swelling. The diffusion and swelling rates are comparable because the rearrangement of polymeric chains occurs slowly while the diffusion process simultaneously causes the time-dependent anomalous effects. Finally, the Super Case II model is characterized by tension and breaking of the poly­mer (solvent crazing).

| **Release exponent (n)** | | | **Drug transport mechanism** |
| --- | --- | --- | --- |
| Cylinder | Sphere | Thin film |  |
| 0.5 | 0.43 | 0.5 | Fickian Diffusion |
| 0.45<n<0.89 | 0.43<n<0.85 | 0.5<n<1 | Anomalous transport  (Diffusion and swelling) |
| 0.89 | 0.85 | 1 | Case I transport  Zero order release  (Polymer swelling) |
| >0.89 | >0.85 | >1 | Super case II transport |

Table 1:Diffusional drug release from polymeric systems [4].

# Baker-Lonsdale Model.

The Baker-Lonsdale model is a modification of the Higuchi model used to describe drug release from spherical matrices [1]–[4], [8]. Mathematically the model is represented by:

|  | $\frac{\boldsymbol{3}}{\boldsymbol{2}}\left[ \boldsymbol{1-}\left( \boldsymbol{1-}\frac{\boldsymbol{M}_{\boldsymbol{t}}}{\boldsymbol{M}_{\boldsymbol{\infty}}} \right)^{\frac{\boldsymbol{2}}{\boldsymbol{3}}} \right]\boldsymbol{-}$ $\frac{\boldsymbol{M}_{\boldsymbol{t}}}{\boldsymbol{M}_{\boldsymbol{\infty}}}\boldsymbol{=}\frac{\boldsymbol{3}\boldsymbol{D}_{\boldsymbol{m}}\boldsymbol{C}_{\boldsymbol{ms}}}{\boldsymbol{r}_{\boldsymbol{0}}^{\boldsymbol{2}}\boldsymbol{C}_{\boldsymbol{0}}}\boldsymbol{t}$ | (28) |
| --- | --- | --- |

Where $M_{t}$ is the amount of drug released at time $t$, $M_{\infty}$ is the amount of drug released at infinite time, $D_{m}$ is the diffusion coefficient, $C_{ms}$ is the drug solubility in the matrix, $r_{0}$is the of the spherical matrix and $C_{0}$ is the initial concentration of the drug in the matrix. Assuming that the initial release of the drug is negligible and re-defining the equation in terms of CFR:

|  | $\frac{\boldsymbol{M}_{\boldsymbol{t}}}{\boldsymbol{M}_{\boldsymbol{\infty}}}\boldsymbol{=CFR}$ | (29) |
| --- | --- | --- |

Equation 28 becomes:

|  | $\frac{\boldsymbol{3}}{\boldsymbol{2}}\left[ \boldsymbol{1-}\left( \boldsymbol{1-CFR} \right)^{\frac{\boldsymbol{2}}{\boldsymbol{3}}} \right]\boldsymbol{-}$ $\boldsymbol{CFR=}\boldsymbol{k}_{\boldsymbol{BL}}\boldsymbol{t}$ | (30) |
| --- | --- | --- |

Where;

$\text{k}_{\text{BL}}\text{=} \frac{\text{3}\text{D}_{\text{m}}\text{C}_{\text{ms}}}{\text{r}_{\text{0}}^{\text{2}}\text{C}_{\text{0}}}\text{ }$

Thus, if $\frac{3}{2}\left[ 1-\left( 1-CFR \right)^{\frac{2}{3}} \right]-$ $CFR$ was plotted against time, $t$, a straight line with slope $k_{BL}$ can be obtained.

Table 2. Summary of kinetic models.

| **Model** | **Equation** | **The equation in terms of Cumulative Fraction Released** | **Assumptions** | **Applications** | **Linearized Form** | **Plot** | |
| --- | --- | --- | --- | --- | --- | --- | --- |
|  |  |  |  |  |  | **x-axis** | **y-axis** |
| Zero-Order | $\frac{dQ(t)}{dt}=K_{0}$ | $CFR=k_{0}t$ | -Dosage forms do not disintegrate.  -Drug released slowly. | Describes the drug dissolution of several types of modified release dosage forms, some transdermal systems and matrix tablets with low soluble drugs in coated forms, osmotic systems, etc. | $CFR=k_{0}t$ | t | CFR |
| First-Order | $\frac{dC(t)}{dt}=-KC\left( t \right)$ | $\ln\left( CFR+ \frac{C_{0}}{C_{T}} \right)=\ln\left( \frac{C_{0}}{C_{T}} \right)-Kt$ | - The change in concentration of the drug in solution is not constant but a function of the instantaneous concentration. | For the dissolution of  pharmaceutical dosage forms such as those  containing water-soluble drugs in porous matrices. | $\ln\left( CFR+ \frac{C_{0}}{C_{T}} \right)=\ln\left( \frac{C_{0}}{C_{T}} \right)-Kt$ | t | ln(CFR) |

| **Model** | **Equation** | **The equation in terms of Cumulative Fraction Released** | **Assumptions** | **Applications** | **Linearized Form** | **Plot** | |
| --- | --- | --- | --- | --- | --- | --- | --- |
|  |  |  |  |  |  | **x-axis** | **y-axis** |
| Higuchi | $Q= k_{h}\sqrt{t}$ | $CFR= \frac{Q-Q_{0}}{Q_{T}}=\frac{k_{h}\sqrt{t}}{Q_{T}}-\frac{Q_{0}}{Q_{T}}$ | - Initial drug concentration must be much higher than drug solubility.  - Diffusivity is constant and occurs only in one dimension  - The size of drug particles is much smaller than the thickness of the film.  - Carrier material does not swell or dissolve.  -Perfect sink conditions. | Describes the dissolution of drug from several types of modified release dosage forms, transdermal systems and matrix tablets with water-soluble drugs. | $CFR= \frac{Q-Q_{0}}{Q_{T}}=\frac{k_{h}\sqrt{t}}{Q_{T}}-\frac{Q_{0}}{Q_{T}}$ | √t | CFR |
| Korsmeyer-Peppas | $CFR\approx k_{kp}t^{n}$ | $\log\left( CFR \right)=\log\left( a \right)+nlog\left( t \right)$ | -The generic equation is applicable for short times.  -M_t_/M_∞_<0.6 used to determine n.  -Drug release occurs in one dimension.  - The system’s length-to-thickness ratio is at least 10. | Describes the drug release from several modified release dosage forms. | $CFR= \frac{M_{t}}{M_{\infty}}=at^{n}$ | log(t) | log(CFR) |

| **Model** | **Equation** | **The equation in terms of Cumulative Fraction Released** | **Assumptions** | **Applications** | **Linearized Form** | **Plot** | |
| --- | --- | --- | --- | --- | --- | --- | --- |
|  |  |  |  |  |  | **x-axis** | **y-axis** |
| Gompertz | $X\left( t \right)=X_{max}\exp\left[ -\alpha e^{\beta\log\left( t \right)} \right]$ | $\ln\left( -\ln\left( CFR \right) \right)=k_{G}\log\left( t \right)+\ln\left( \alpha\right)$ | -Assume good stability and an intermediate release rate. | To describe in-vitro dissolution profiles. | $\ln\left( -\ln\left( CFR \right) \right)=k_{G}\log\left( t \right)+\ln\left( \alpha\right)$ | log(t) | ln[-ln(CFR)] |

**Abbreviations**: ***t***, time; ***CFR***, Cumulative fraction released.


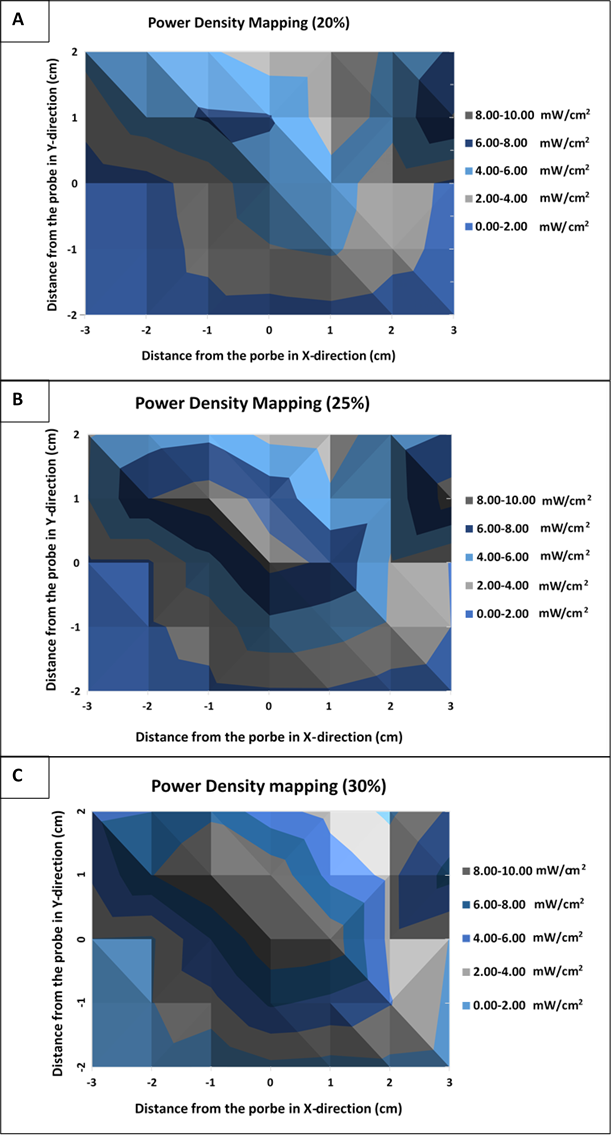


Figure 1. Power intensity values at various distances from the probe at 20% amplitude (A), 25% (B) and 30% (C).

# References

[1] P. Costa and J. M. Sousa Lobo, “Modeling and comparison of dissolution profiles,” *Eur. J. Pharm. Sci.*, vol. 13, no. 2, pp. 123–133, May 2001, doi: 10.1016/S0928-0987(01)00095-1.

[2] M. P. Paarakh, P. A. N. I. Jose, C. M. Setty, and G. V Peter, “Release Kinetics – Concepts and Applications,” *Int. J. Pharm. Res. Technol.*, vol. 10, no. 1, pp. 1–9, 2018.

[3] R. A. Siegel and M. J. Rathbone, “Fundamentals and applications of controlled release drug delivery,” *Adv. Deliv. Sci. Technol. ed. J. Siepmann al., Control. Release Soc.*, 2012.

[4] M. L. Bruschi, “Mathematical models of drug release,” in *Strategies to Modify the Drug Release from Pharmaceutical Systems*, Woodhead Publishing, 2015, pp. 63–86.

[5] S. Dash, P. N. Murthy, L. Nath, and P. Chowdhury, “Kinetic modeling on drug release from controlled drug delivery systems.,” *Acta Pol. Pharm.*, vol. 67, no. 3, pp. 217–23.

[6] N. A. Peppas, “Analysis of Fickian and non-Fickian drug release from polymers.,” *Pharm. Acta Helv.*, vol. 60, no. 4, pp. 110–111, Jan. 1985.

[7] N. A. Peppas and B. Narasimhan, “Mathematical models in drug delivery: How modeling has shaped the way we design new drug delivery systems,” *Journal of Controlled Release*, vol. 190. Elsevier B.V., pp. 75–81, 28-Sep-2014, doi: 10.1016/j.jconrel.2014.06.041.

[8] N. Shahrin, “SOLUBILITY AND DISSOLUTION OF DRUG PRODUCT: A REVIEW,” *Int. J. Pharm. Life Sci.*, vol. 2, no. 1, pp. 33–39, 2013.
